# Impact of Different Enzymatic Processes on Antioxidant, Nutritional and Functional Properties of Soy Protein Hydrolysates Incorporated into Novel Cookies

**DOI:** 10.3390/foods12010024

**Published:** 2022-12-21

**Authors:** Zorica Knežević-Jugović, Alina Culetu, Jelena Mijalković, Denisa Duta, Andrea Stefanović, Nataša Šekuljica, Verica Đorđević, Mirjana Antov

**Affiliations:** 1 Department of Biochemical Engineering and Biotechnology, Faculty of Technology and Metallurgy, University of Belgrade, 11000 Belgrade, Serbia; 2 National Institute of Research & Development for Food Bioresources—IBA Bucharest, 021102 Bucharest, Romania; 3 Innovation Center of Faculty of Technology and Metallurgy, 11000 Belgrade, Serbia; 4 Department of Chemical Engineering, Faculty of Technology and Metallurgy, University of Belgrade, 11000 Belgrade, Serbia; 5 Department of Applied and Engineering Chemistry, Faculty of Technology, University of Novi Sad, 21000 Novi Sad, Serbia

**Keywords:** soy protein concentrate, proteases, antioxidant activity, amino acid profile, rheological properties, cookie formulation

## Abstract

Soy protein concentrate (SPC) was hydrolyzed using several commercial food-grade proteases (Alcalase, Neutrase, papain, Everlase, Umamizyme, Flavourzyme) and their combination to obtain promising ingredients in the manufacture of functional bakery products. In all cases, the hydrolysis caused nutritional, sensory, and rheological changes in SPC, as well as protein structural changes like increased surface hydrophobicity and content of exposed SH groups with the magnitude of these changes depending on enzyme specificity. The hydrolysis with the combination of Neutrase and Flavourzyme (NeuFlav) increased essential amino acid content by 9.8% and that of Lys by 32.6% compared to SPC. This hydrolysate showed also significant antioxidant activities including ABTS and superoxide anion scavenging activity and metal-chelating ability. The addition of all hydrolysates in wheat flour decreased water adsorption and increased development time to some extent due to gluten network weakening, but also decreased the rate of starch retrogradation, contributing to the increase of the shelf-life of bakery products. The NeuFlav tasted less bitter than other hydrolysates, while E-nose provided a discrimination index of 93 between control and hydrolysates. It appeared that the addition of the NeuFlav hydrolysate in a cookie formulation improved protein content and nutritional quality and directed to its higher general consumer acceptability than cookies formulated with only wheat flour.

## 1. Introduction

Soy proteins are widely used in several food products in the forms of soy flour, soy protein concentrate (SPC), and soy protein isolate (SPI) to improve nutritional content and achieve desired functional properties like increased water absorption, fat binding, viscosity, gelation, foaming, emulsification, and other favorable properties [1,2]. A good way to incorporate soy proteins into food products increasing their nutritional value is represented by cookies, which are baked goods appreciated by many people, especially children, traditionally made by using wheat flour or other types of flour, sugar, and fat [3,4]. For example, soy protein isolate was used in cookies formulations to enhance their nutrient values, with a maximum level of addition of 15–20% from the consumers’ acceptability point of view [5,6]. However, recent research efforts in this field also continue to look at novel functional properties and applications of soy proteins through physical, chemical, or enzymatic modifications [7,8]. In that sense, the production of hydrolyzed soy protein, which, in addition to being excellent source of nutrients, can be used as a source of peptides exerting biological functions, particularly antioxidant activity, has received increasing attention [9]. Among several different technologies for soy protein hydrolysate production, the use of enzymes is a suitable option because of mild reaction conditions and specific action enabling producers to precisely control the hydrolytic reaction and tailor the functional properties of the final product [10,11,12].

Research focusing on bioactive peptides released through the enzymatic hydrolysis of soy protein has intensified in recent years, particularly due to an increasing need to replace commonly used synthetic antioxidants in the food industry like butylated hydroxytoluene or butylated hydroxyanisole with nontoxic natural counterparts, as well as animal proteins with plant-derived proteins. It seems that enzyme type and hydrolysis conditions are key parameters in the process for determining bioactive and functional properties of hydrolysates that are related to changes in protein structure, reduction in molecular size, and the surface exposition of polar and hydrophobic groups [11,13]. Thus, depending on protein source pretreatment, enzymes used, and processing conditions, the antioxidant activity of the obtained hydrolysates is quite different. For example, native and heated SPI hydrolyzed with different enzymes including pepsin, chymotrypsin, papain, Alcalase, Protamex, and Flavourzyme resulted in peptide mixtures with different inhibitory effects on lipid oxidation ranging from 28 to 65% in phosphatidylcholine liposome model system [14]. Castro and Sato [11] also observed differences in the DPPH and ORAC radical-scavenging capacity of SPI hydrolysates when different microbial proteases were used, reporting a 7.0-fold increase in antioxidant activity after hydrolysis using Flavourzyme. Coscueta et al. [15] identified peptides from soy protein with antioxidant and antihypertensive activities obtained for the first time with the commercial enzyme Corolase PP. Other microbial proteases like neutral protease from *Bacillus subtilis*, Validase from *Aspergillus oryze*, and alkaline protease from *B. licheniformis* can be useful to generate soy protein hydrolysates with improved antioxidant properties [16]. In silico enzymatic analysis conducted on the selected soy glycinin G4 subunits B3, A4, and A5, revealed that proteinase K showed great feasibility to release peptides with high antioxidant activity, while cathepsin G, glutamyl endopeptidase II, glycyl endopeptidase, chymosin, or ginger protease did not produce any bioactive peptides from A4 polypeptide [17]. However, one of the limitations of this in silico investigation is that the prediction of bioactive peptides liberated from selected proteins did not incorporate factors such as pH, temperature, and duration of hydrolysis. Furthermore, most current research is focused on SPI, and examined samples come from this substrate [7,10,13]. There is a general lack of knowledge about the effects of protease hydrolysis on soy products other than SPI, such as soy flour or SPC.

The use of cheap raw materials of lower commercial value such as SPC instead of SPI can reduce the number of technological operations, energy, and material requirements in the process, simultaneously reducing production costs. However, few studies have reported the beneficial effects of protease hydrolysis on soy products other than SPI, such as soy flour, SPC, or soy sauce cake extruded soy protein concentrate even if the outcomes of such studies are more relevant for potential industrial application [17,18]. Furthermore, the number of enzymes tested is limited to just a few including bromelain [18,19] and therefore, there is a lack of information concerning the effects of enzyme specificity and the design of the enzymatic process on the physicochemical, nutritional, rheological, and antioxidant properties of the obtained hydrolysates, particularly the potential application in the bakery industry.

Therefore, for the production of soy protein ingredients to be more feasible in the industrial application, we used SPC containing around 70% dw (*N* × 6.25) protein as the substrate. The aim was to investigate the effects of several commercial food-grade proteases on the protein yield, nutrition composition, antioxidant activity, sensory, and rheological properties of the obtained hydrolysates and provide more information regarding their features for potential application in the bakery industry. The results are discussed within the context of the bakery industry but could also be of general relevance to the food industry.

## 2. Materials and Methods

### 2.1. Raw Material and Enzymes

Soy protein concentrate (SPC) (commercial name TRADCON F200) with the moisture content of 5.81%, crude protein content of 70.96 ± 1.77% dw (*N* × 6.25), ash content of 6.41 ± 0.51%, fat content of 0.061 ± 0.032, cellulose content of 3.80 ± 0.18%, and total fiber content of 20.63 ± 1.44% was a kind gift from the SOJAPROTEIN d.o.o. (Bečej, Serbia) and used as a substrate (granulation: min 90% < 0.075 mm).

The proteases used in the hydrolysis were: Alcalase^®^ 2.4 L (EC 3.4.21.62, endoprotease from *Bacillus licheniformis*), Neutrase^®^ 0.8 L (EC 3.4.24.28, neutral, zinc metallo-endoprotease from *B. amyloliquefaciens*), Everlase^®^ 8.0 L (detergent endoprotease from *Bacillus* sp. GMO engineered), and papain from *papaya latex* (EC 3.4.22.2, cysteine protease), with the claimed activities of 2.4 Anson Unit (AU)/g, 0.8 AU/g, 8 U/g, and 1.5–10 AU/mg_solid_, respectively, all purchased from Sigma Aldrich Co. (St. Louis, MO, USA), and Umamizyme (mixture of endoprotease and exopeptidase from *Aspergillus oryzae*) from Amano Enzyme Inc. (Nagoya, Japan) with the declared activity of 74.3 U/g. In the two-step enzymatic processes, Flavourzyme (EC 3.4.11.1), a mixture of endoprotease and exopeptidase from *A. oryzae* (Sigma Aldrich Co., St. Louis, MO, USA) was used.

### 2.2. Chemicals

2,2-Azino-bis(3-ethylbenzothiazoline-6-sulfonic acid (ABTS), 2,4,6-trinitrobenzene sulfonic acid (TNBS), bovine serum albumin (BSA), 8-anilino-1-naphthalene (ANS), potassium persulfate, tris(hydroxymethyl)aminomethane (Tris), urea, sodium dodecylsulfate (SDS), dithiothreitol (DTT), glycine, EDTA, standards of amino acids, and other chemicals were purchased from Sigma Aldrich Co (St. Louis, MO, USA). All other chemicals used were of analytical grade and are identified in the text. The deionized water used to prepare samples and eluents (18.2 MΩ) was generated using a Milli-Q purification system (Merck Millipore Advantage A10, Darmstadt, Germany).

### 2.3. One-Step and Two-Step Enzymatic Hydrolysis

The enzymatic hydrolysis conducted in the batch-stirred reactor consisted of a 400 mL glass vessel with a water jacket, a mechanical stirrer equipped with the four-bladed propeller, and a pH electrode according to the previously published protocol with slight modification [20]. The reaction was performed as a one-step or two-step process using six food-grade commercial proteases as follows. SPC was dispersed in deionized water to obtain 8% solution *w*/*v* (based on protein content, *N* × 6.25), and equilibrated to the optimal pH and temperature for each enzyme. The protease was added in 200 mL of the reaction mixture at two enzyme/substrate ratios (1.0% and 4.0% *w*/*w*, enzyme mass/protein mass), and the mixture was stirred at a constant rate (200 rpm) maintaining optimum conditions (for Alcalase: pH 8, T = 55 °C; papain: pH 8, T = 50 °C; Neutrase: pH 7, T = 45 °C; Umamizyme: pH 8, T = 50 °C; Everlase: pH 9, T = 60 °C). The process conditions used were based on the protease activity and preliminary study. For two-step processes, following the hydrolysis with an endoprotease (Alcalase or Neutrase) for the first 75 min, the mixture was equilibrated to 50 °C and pH 7 using 1 M HCl and Flavourzyme was added at an *E*/*S* ratio of 4% *w*/*w* for another 120 min. The reaction was stopped by heating at 90 °C for 15 min, and the hydrolysates were rapidly cooled to 25 °C, and the insoluble part was removed by centrifugation at 10,000× *g* for 10 min and 4 °C (Heraeus™ Fresco™ 17 Microcentrifuge, Thermo Fisher Scientific, Waltham, MA, USA). The supernatants (soluble fraction) were collected and stored at −20 °C or spray-dried by using a Mini Buchi B-290 spray-dryer (BÜCHI Labortechnik AG, Flawil, Switzerland) under the following conditions: input and output temperature 160 °C and 90 °C, nozzles → 5, compressed air flow for liquid dispersion of 800 L/h, sample flow 8.5 mL/min. Dried hydrolysates were weighed and analyzed for protein content and further analysis. Seven batches of hydrolysate produced with Alcalase, Everlase, Neutrase, papain, Umamizyme, Alcalase/Flavourzyme, and Neutrase/Flavourzyme were labeled Alc, Ever, Neu, Pap, Umam, AlcFlav, and NeuFlav. The hydrolysis was done in triplicate for each condition.

### 2.4. Degree of Hydrolysis and Protein Recovery

The degree of hydrolysis (DH) defined as the percentage of peptide bonds cleaved was calculated based on the consumption of base (0.1 M NaOH) by using the pH-stat method of Adler-Nissen described by Stefanović et al. [21] according to the following equation:

(1) $$DH\;\left(\%\right)=\frac{N_{b}\cdot B\cdot 100}{\alpha \cdot m_{p}\cdot h_{tot}}$$

where *N_b_* is the normality of the base, *B* is base consumption (mL), *h_tot_* is the total amount of peptide bonds per weight unit of the protein (for soy protein is 7.8 mmol/g), *α* is the degree of dissociation of the *α*-amino groups and *m_p_* is the mass of protein (g).

The free amino group content of the samples was measured at different time intervals by the TNBS spectrophotometric method, and taken as a measure of the *DH* [22]. The absorbance was measured at 420 nm and *α*-amino acid was expressed in terms of *L*-leucine. Good correlations between TNBS and pH-stat method were found.

The protein recovery, *PR* was calculated as the ratio of the protein content of the sample after hydrolysis to the protein content of the non-hydrolyzed sample using the equation:

(2) $$PR\left(\%\right)=\frac{C_{h}\cdot m_{h}}{C_{\mathrm{SPC}}\cdot m_{\mathrm{SPC}}}\cdot 100$$

where *C_h_* and *m_h_* are protein content (mg/g), and mass of the obtained hydrolysates (g), *C*_SPC_ and *m*_SPC_ are protein content (mg/g) and mass of SPC (g) used for hydrolysis, respectively.

### 2.5. Determination of Protein Content

Crude protein (*N* × 6.25) in SPC and hydrolysates powder was determined by the Kjeldahl method [23], and the protein concentration of hydrolysate solutions was measured by the method of Lowry et al. [24] using BSA as a standard.

### 2.6. Surface Hydrophobicity Measurement

The relative fluorescence as a measure of the protein surface hydrophobicity was determined with ANS as a probe according to Mu et al. [25] with minor modification. Briefly, the samples were diluted with 0.1 M phosphate buffer (pH 7) to obtain a range of protein concentration of 0.01–1.0 mg/mL and poured into quartz cuvettes. The fluorescence intensity was measured at 25 °C using a Horiba FluoroMax-4 spectrofluorometer (Kyoto, Japan) at the excitation and emission wavelengths of 365 and 480 nm, with a constant excitation and emission slit of 2.5 and 10 nm/s of scanning speed. After the addition of 7 µL of 8.0 mM ANS in phosphate buffer to 1.393 mL of protein solution, the fluorescence intensity was read. The index of surface aromatic hydrophobicity, *H*_0_ was expressed as the initial slope of the plot of fluorescence intensity as a function of protein concentration.

### 2.7. Determination of Sulfhydryl (SH) Groups

The content of SH groups was determined according to Elman’s procedure using 5,5-dithiobis-(2-nitrobenzoate), DTNB, with minor modification, as described by Stefanović et al. [21]. The content of reactive (exposed) SH groups was determined as follows. Samples’ protein content was adjusted to a concentration of 1% (w w-1) with a buffer pH 8.0 consisting of 86 mM TRIS, 90 mM glycine, 4 mM EDTA, and centrifuged for 20 min at 12,300× *g*. An amount of 0.025 mL of Ellman’s reagent (4 mg/mL) was added to 2.5 mL of supernatants, followed by gentle stirring for 15 min at room temperature. The absorbance was measured at 412 nm against a reagent blank. The content of total SH groups was determined following the same protocol, but using denaturing buffer consisting of 86 mM TRIS, 90 mM glycine, 4 mM EDTA, 8 M urea, and 0.5% (*w*/*v*) SDS.

### 2.8. Analysis of Amino Acid Composition

The amino acids composition was determined by high performance anion-exchange chromatographic technique coupled with integrated pulsed amperometric detection (HPAEC-IPAD) at the standardized laboratory of SOJAPROTEIN. These experiments were conducted using the Dionex AAA-Direct system (Thermo Scientific, San Jose, CA, USA) with a Dionex™ ICS-5000+ ED Electrochemical Detector using a pH, Ag/AgCl reference electrode, and a gold AAA-Direct disposable working electrode, also purchased from Dionex. The anion-exchange column AminoPak PA10 (2 × 250mm, Dionex, Sunnyvale, CA, USA) and its guard column (AminoPak PA10 guard, 2 × 50 mm, Dionex) were kept at 30 °C and the injection volume was set to 25 µL. The samples were first hydrolyzed at 110 °C for 24 h with 6 M HCl containing 1% (*w*/*v*) phenol, filtered through 0.22 µM filter (Millex-GP, polyethersulfone, Millipore, Burlington, MA, USA), and amino acid separation was performed at a flow rate of 0.25 mL min^−1^ under the gradient conditions as reported by Lamberts et al. [26]. Cysteine is thereby oxidized to cystine. Amino acid content was expressed on dry matter protein. Amino acid score (AAS) was calculated for each essential amino acid by dividing its content by the reference value for the amino acid.

### 2.9. Determination of Antioxidant Activity

#### 2.9.1. Determination of the ABTS^•+^ Radical Scavenging Activity

The method was based on the ABTS^•+^ radical cation decolorization [27]. Briefly, the radical cation was obtained in the reaction between 7 mM ABTS solution and 2.45 mM potassium persulfate, left in dark for 12–16 h, and then diluted with 5 mM phosphate buffer, pH 7.4, until the absorbance of 0.70 ± 0.02 was achieved. An aliquot of the sample (5 µL) with protein content adjusted to 5 mg/mL was added into 500 µL of prepared ABTS^•+^ solution and after 5 min, absorbance was measured at 734 nm. The scavenging activity was calculated as follows:

(3) $$ABTS\;\left(\%\right)=\left(1-\frac{A_{s}}{A_{c}}\right)\cdot 100$$

where *A_s_* is the absorbance of the sample and *A_c_* is the absorbance of the control.

#### 2.9.2. Determination of the Metal Ion Chelating Activity (MICA)

The method of Decker and Welch was [28] used with slight modification. An aliquot of 0.20 mL of samples with protein content adjusted to 5 mg/mL was pre-mixed with 0.80 mL of double distilled water and 0.1 mL of 2 mM FeCl_2_, intensively vortexed and incubated at 25 °C for 3 min. Then, 0.10 mL of 5 mM ferrozine solution was added and the mixture was vortexed and kept further at 25 °C for 10 min. The absorbance of the resulting solution was measured at 562 nm. The activity (%) was calculated as follows:

(4) $$MICA\;\left(\%\right)=\left(1-\frac{A_{s}}{A_{c}}\right)\cdot 100$$

where *A_s_* is the absorbance of the sample and *A_c_* is the absorbance of the control.

#### 2.9.3. Determination of the Superoxide Radical Scavenging (SRS) Activity

The method is based on spectrophotometric monitoring of the inhibition of pyrogallol autoxidation as described by Xie et al. [29] with slight modifications. Briefly, the samples were diluted with 50 mM TRIS-HCl buffer containing 1 mM EDTA (pH 8.3) to a final concentration of 5 mg/mL, and an aliquot of 0.16 mL was conveyed into a clear microplate well. After that, 0.16 mL of 1.5 mM pyrogallol solution (Sigma Aldrich) was put into each well, and the plate was incubated at 25 °C in the dark for 8 min. The absorbance was measured at 320 nm immediately after the addition of the pyrogallol over 8 min using a microplate reader (Multiscan GO, Thermo Fisher Scientific, Waltham, MA, USA). The *SRS* activity was calculated as follows:

(5) $$SRS\;\left(\%\right)=\frac{{\left(\frac{\Delta A}{min}\right)}_{b}-{\left(\frac{\Delta A}{min}\right)}_{s}}{{\left(\frac{\Delta A}{min}\right)}_{b}}\cdot 100$$

where *b* and *s* are blank and sample, respectively.

### 2.10. Characterization of Sensory Properties of Hydrolysates

#### 2.10.1. Determination of Color

The color was determined by a bench-top Konica Minolta CM-5 spectrophotometer with a D65 illuminant, a 10° observer angle, and SpectraMagicTM NX software (Konica Minolta Sensing, Inc., Osaka, Japan). Before measurements, the instrument was calibrated against black and white tiles. Samples were homogeneously poured into glass Petri dishes and air bubbles were removed. The results were expressed as L* (lightness), a* (−*a* = green, +*a* = red), and b* (−*b* = blue, +*b* = yellow) and reported as the mean of ten measurements at different points by rotating the sample cup.

#### 2.10.2. Determination of Bitterness

The bitterness of the samples was analyzed using a paired comparison test, ISO 5495, [30] with a panel of 20 evaluators selected according to ISO 8586 [31]. The assay was conducted in a standardized sensory analysis room, according to ISO 8589 [32]. Samples were mixed and stirred with water to prepare 1% solutions. Each panelist was presented with a pair of samples consisting of a reference, R (commercial soy protein hydrolysate, widely used in the food industry, provided by A. Constantino & C. S.p.A, Favria, Italy), and each one of the hydrolysates codded with 3 random letters. Each pair of samples was served to the panel in a random order at room temperature in plastic cups. The samples were monadic and evaluated in 4 sessions. The evaluators were instructed to assess bitterness. The intensity of bitterness was rated using a 5-point bipolar scale as follows: samples with a lower degree of bitterness than R (marked negatively), samples similar with R (marked with 0), and samples with a much higher degree of bitterness than R (marked positively). Thus, the sum of classes was calculated for each sample.

#### 2.10.3. Headspace-Electronic Nose Analysis of Volatile Compounds

The headspace of samples was investigated with an electronic nose system combined with HS100 auto-sampler together with α Soft software for data processing (Alpha M.O.S.—model FOX 4000, Toulouse, France). An amount of 0.5 g of hydrolysate was weight in a 10 mL vial, hermetically sealed with a PTFE/silicone septum and incubated for 900 s at 70 °C under agitation (250 rpm) to allow the volatilization of compounds into the headspace. Synthetic air and nitrogen were used as carrier gas with a flow of 150 mL/min. The volume of injection of the SPH headspace into the measuring chamber of the electronic nose was 2500 µL, with an acquisition time of 120 s. All samples were run in triplicate and the individual signals recorded were used for statistical analysis.

### 2.11. Mixolab Analysis of Rheological Properties

For each type of hydrolysate, three mixtures were prepared by mixing the wheat flour and hydrolysate in the proportions of 95:5 (*w*/*w*). Before use, the mixtures were homogenized for 15 min in a planetary mixer (Krups PowerMix, type 417, Paris, France). The rheological behavior was evaluated using a Mixolab analyzer (Chopin Technologies, Villeneuve-la-Garenne, France) with the “Chopin^+^” protocol [33]. The Mixolab measures the torque (in Nm) produced by dough between two kneading arms and temperature changes, to simulate the bread-making process. The following settings were used: initial mixing for 8 min at 30 °C, heating to 90 °C with 4 °C 1/min, holding for 7 min at 90 °C, cooling to 50 °C with 4 °C 1/min and maintaining for 5 min at 50 °C. The mixing speed was kept constant at 80 rpm and the total time of analysis was 45 min. The analysis was repeated 3 times for each sample. Wheat flour was used as a control. Parameters obtained from the recorded curves and calculated by Mixolab software (version 4.0.8) were: water absorption (amount of water required to obtain the maximum torque), development time (time required to reach the maximum torque), stability (mixing resistance of dough), C2 (torque associated with protein weakening based on mechanical work and temperature increase), C3 (rate of starch gelatinization), C4 (stability of the starch gel formed), C5 (represents starch retrogradation during the cooling period).

### 2.12. Preparation of Cookies and Chemical Analysis

Cookies were manufactured using white wheat flour as the base ingredient and the sample made with 100% wheat flour was the control. The cookie formulation was: wheat flour (1 kg), white sugar (475 g), sunflower oil (775 mL), eggs (8 pcs), and ammonium bicarbonate (4 g). For sample cookies, wheat flour was substituted with 5% hydrolysates. All the ingredients were purchased from a local market and the cookies were prepared according to the method described by Duta and Culetu [34]. The chemical composition of the cookies was determined in duplicate according to the standard methods reported in a previous study [35]. For the overall volatile composition of the cookies by electronic nose system, the method described by Duta and Culetu [34] was employed.

### 2.13. Statistical Analysis

All experiments were done in duplicate or triplicate and results were expressed as mean ± standard deviation. The results were compared by one-way analysis of variance ANOVA (Minitab^®^17 software, Minitab Ltd., Coventry, UK). Tukey test was applied for comparing differences between mean values at a 95% confidence level (*p* < 0.05).

## 3. Results and Discussion

### 3.1. Enzymatic Hydrolysis of SPC

**Figure 1:**
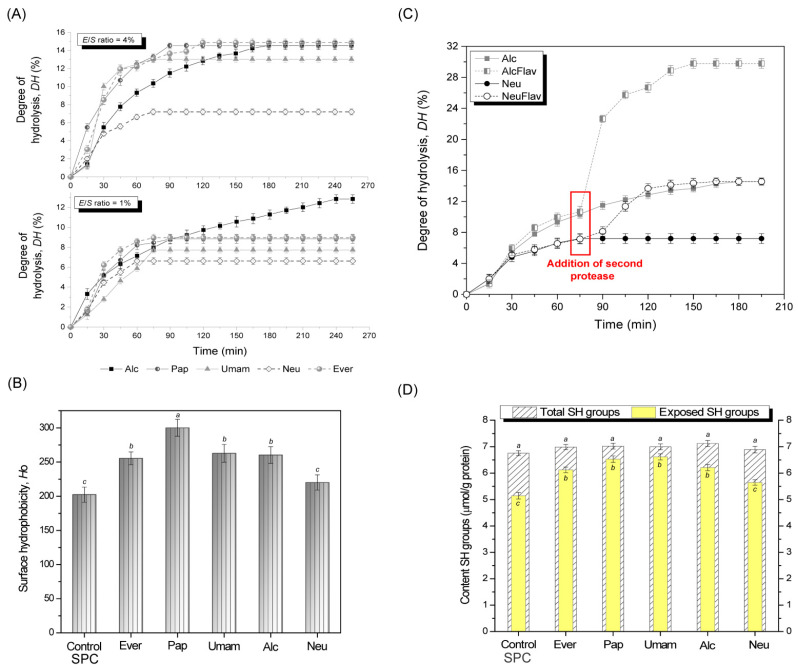
Kinetics of SPC hydrolysis with (**A**) Alcalase, Neutrase, papain, Everlase and Umamizyme alone at two *E*/*S* ratios; and (**B**) with Alcalase or Neutrase (added at time 0 min) and Flavourzyme (added after 75 min) in the two-step enzymatic process; (**C**) surface hydrophobicity and (**D**) content of SH groups of SPC (control) and hydrolysates prepared with different enzymes. Data correspond to the average SD of three (**A**,**B**) and two (**C**,**D**) determinations. Results are expressed as means ± standard deviation (n = 2 (for graphs **A**,**B**) and n = 3 (for graphs **C**,**D**)). Means with different lower letters in the same graph are significantly different (*p* < 0.05).

The five proteolytic enzymes (Alcalase, Neutrase, papain, Everlase and Umamizyme) used for hydrolysis of SPC led to different reaction kinetics resulting in hydrolysates of variable degree of hydrolysis (*DH* from 7.2 to 14.9%), as presented in Figure 1A. Generally, the reaction rate and *DH* obtained by Alcalase, papain and Everlase were higher than those obtained with other two enzymes tested at both *E*/*S* ratios. Except for the Alcalase-catalyzed reaction, all reactions progressed rapidly for the first 30 min and then relatively slowly reaching a plateau between 60 and 90 min, depending on enzyme and *E*/*S* ratio used. The Alcalase-catalyzed reaction steadily progressed over time, necessitating longer processing time (180 min at *E*/*S* ratio of 4%) to reach a similar *DH* of 14.5% as with papain or Everlase. The *DH* showed significant increase (*p* < 0.05) with increased *E*/*S* ratio from 1 to 4% for all proteases evaluated, thus the *E*/*S* ratio of 4% was selected for further investigation.

When comparing *DH* obtained with different enzymes, it appeared that the soy protein showed a higher susceptibility toward papain, Everlase, or Alcalase compared to Neutrase or Umamizyme. At a higher *E*/*S* ratio of 4%, the maximum determined *DH* with Neutrase reached the value of 7.2 ± 0.63% after 75 min of hydrolysis, and the remaining peptide fragments appeared to be resistant to further proteolytic degradation even after 255 min, suggesting that the majority peptide bonds were sterically inaccessible to the enzyme. The results and the kinetic curve shape are in accordance with the results obtained for the Neutrase-catalyzed hydrolysis of other proteins from rawhide or pig bones [35,36]. Namely, Neutrase is a metalloprotease having an endopeptidase activity cleaving randomly internal peptide bonds, but the protein degradation appears to start from the exterior since the enzyme absorbs tightly at the surface. Thus, the interior peptide bonds become accessible to the enzyme only in the course of progressive degradation from the outside. Consequently, the addition of Flavourzyme with both endo- and exopeptidase activity to the Neutrase/SPC reaction mixture after 75 min had a significant positive effect on the hydrolysis, increasing the *DH* by 2-fold (Figure 1B). This could be due to the exopeptidase activity of Flavourzyme which also cleaved peptide bonds next to the end of the polypeptide chains and caused the opening of the chains, allowing the Neutrase to access the cleavage sites. The combined utilization of enzymes with dissimilar specificity also improved the reaction rate of the Alcalase-catalyzed reaction, increasing the final *DH* from 14.55 ± 0.50 to 29.78 ± 0.62%.

Enzymatic hydrolysis also caused structural changes in the soy protein, such as increased hydrophobic surface area and content of exposed SH groups (Figure 1C,D). These parameters are factors of prime importance since they provide information on the partial unfolding of protein and may have crucial effects on further functional or sensory properties like bitterness.

In agreement with previous reports, all hydrolysates presented higher surface hydrophobicity compared to SPC [37,38]. The hydrolysates Alc, Ever, and Umam presented similar fluorescence emission spectra, while the Pap hydrolysate showed much more intensive fluorescence emission spectra, revealing an increase in protein flexibility and exposure of aromatic amino acids to the solvent. No significant difference in the content of total SH groups among all hydrolysates was noticed, regardless of the enzyme used, but the content of exposed SH groups was different. The hydrolysates Pap and Umam presented higher content of exposed SH groups compared to control and other hydrolysates, whereas the Neu hydrolysate showed the lowest. The Neu showed the lowest surface hydrophobicity, revealing that the increase in the surface hydrophobicity was accompanied by an increase in the content of exposed -SH groups, which are both indicative of the level of protein unfolding and peptide chain flexibility. The limited and controlled proteolysis disrupted the protein tertiary structure and unfolded the protein chains, exposing the SH and hydrophobic groups, with the magnitude of these changes depending on enzyme specificity and a corresponding peptide sequence and length. It is important to relate the structure of the proteins with functional properties for predicting their suitability in food processing. It appeared that the solubility of all samples increased significantly after hydrolysis when compared with untreated SPC (Supplementary Materials, Figure S1). The solubility of the Pap hydrolysate was the highest, suggesting that the solubility was positively correlated with both surface hydrophobicity and the content of exposed SH groups. This behavior is consistent with that observed by Hu et al. [39], who reported that SPI solubility was promoted by greater surface hydrophobicity.

### 3.2. Amino Acid Composition

For soy protein hydrolysate intended for food application good taste and nutritional properties are important. Unfortunately, the hydrolysis often results in a low yield of essential amino acids and bitter taste. The protein recovery and amino acid content of the hydrolysates obtained with different enzymes in one and two-step processes are presented in Table 1.

The enzymatic hydrolysis seemed to increase the protein content of the soluble portion from SPC and depleted the protein in the remaining insoluble fraction. The percentage of the soluble protein recovered from SPC after hydrolysis varied from 68.59 to 82.19%, depending on the enzyme used. The protein recovery with papain was 78.17%, which is significantly higher than with other enzymes, revealing that papain is most suitable to solubilize SPC. On the other hand, Alcalase and neutral proteases were less appropriate. However, the combined enzymatic hydrolysis with Alcalase and Flavourzyme improved the protein recovery due to the combination of the broad endoprotease activity of Alcalase and endo- and exoprotease activities of Flavourzyme.

The quantitative determination of amino acids in the hydrolysates revealed that soy protein hydrolysates, after separation of the insoluble fraction, differed significantly in nearly all evaluated amino acid content with respect to SPC, particularly arginine, lysine, alanine, threonine, tyrosine, aspartic and glutamic acids. Thus, depending on enzyme specificity, the obtained hydrolysates even at similar DH seemed to have different protein and peptide profiles regarding size and sequence affecting their solubility and, therefore, the soluble fraction had different amino acid profiles.

It appeared that all hydrolysates as well as SPC did not contain any limiting essential amino acids (EAA) since all AAS values were higher than 1 (Table 1), which was consistent with the fact that soy protein isolate, and concentrate have been regularly reported as good quality proteins [41]. Apparently, the enzymatic hydrolysis improved the content of several amino acids, especially lysine which is deficient in most cereal grains. The content of Lys increased significantly in the case of Pap or Umam hydrolysate, but also in the hydrolysates, AlcFlav and NeuFlav obtained in two-step enzymatic processes, which content could be enough to meet Lys requirements even for infants. The total amount of EAA (histidine, threonine, valine, methionine, phenylalanine, isoleucine, and leucine) was the highest in the hydrolysates NeuFlav (39.13 g/100 g protein) and AlcFlav (38.83 g/100 g) obtained by the enzyme combination, but it was also rather high in the Pap hydrolysate (38.37 g/100 g). In the latter, high levels of lysine, tyrosine, phenylalanine, valine, and proline were apparent. This could be due to papain specificity since it has been shown to cleave basic or positively charged amino acids such as arginine, histidine, and lysine, along with residues that follow phenylalanine [42]. Generally, SPC lacked sulfur-containing amino acids methionine and cysteine. According to FAO/WHO, the requirements for sulfur-containing amino acids for preschool children and infants are 2.5 and 4.2 g/100 g of protein, while those for Phe+Tyr are 6.30 and 7.2 g/100 g, respectively. It appeared that the Met+Cystine content in all hydrolysates would meet the requirements for children and adults, while Phe+Tyr content, even for infants [43]. In addition, Leu and His recommendations for infants could be supplied by NeuFlav alone, revealing that the combination of endo- and exoprotease activities of Neutrase and Flavourzyme improved the soy protein’s nutritional quality.

### 3.3. Antioxidant Activity of SPC Hydrolysates

**Figure 2:**
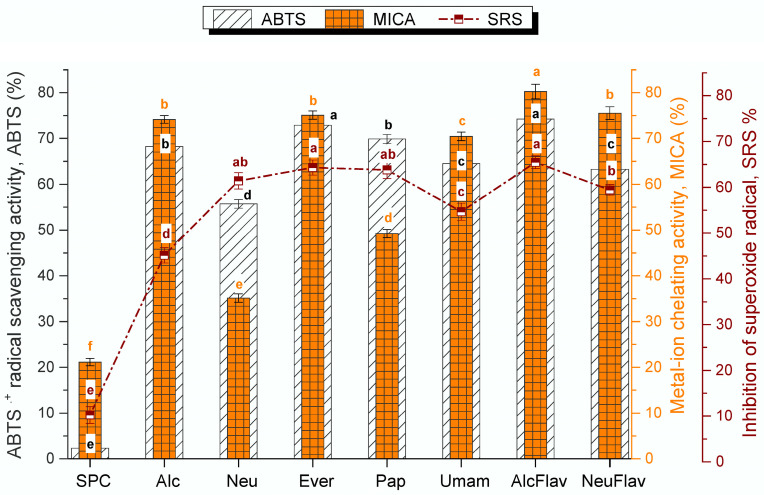
Antioxidant activity of SPC hydrolysates obtained with different enzymes in one-step and two-step processes. Data expressed as a mean ± STD of three independent experiments. Samples sharing the same letter are not significantly different from each other at level 0.05.

The enzymatic hydrolysis of food proteins is carried out not only for the improvement of the nutritional and functional properties but also for the production of hydrolysates with improved antioxidant capacity. The antioxidant properties could be attributed to the combined effect of a number of properties concerning their ability to eliminate free radicals, donate electrons or chelate metal ions [44]. Here, we tested the antioxidant activity in terms of ABTS scavenging activity, superoxide radical (O_2_^•−^) scavenging activity (*SRS* activity), and the ability to chelate metal ions (MICA). The results are presented in Figure 2.

The enzymatic hydrolysis in all cases significantly enhanced the antioxidant activity, which was confirmed by three methods. Among five single enzymes, the hydrolysates Ever and Pap had the highest ABTS activity (72.86 ± 0.98 and 69.86 ± 0.97%, respectively), whereas the Neu hydrolysate showed the lowest of 59.44 ± 1.21%. These ABTS activities are higher or comparable to value reported by Guan et al. for soybean-protein hydrolysates obtained from SPI by combining high pressure and Corolase PP treatment [45]. As to *SRS* activity, the hydrolysates Ever, Pap and Neu showed similar *SRS* activity (64.31 ± 0.91, 63.79 ± 0.91 and 61.43 ± 1.78), but statistically higher to those of Alc (45.16 ± 1.69) or Umam hydrolysate (54.68 ± 1.86%).

Among hydrolysates obtained with single enzyme, the most effective in ability to chelate metals were Alc and Ever hydrolysates, followed by Umam and Pap. Although the Alc hydrolysate did not show a strong *SRS* activity, it exhibited considerable ability to scavenge ABTS radical or to chelate metal ion. On the other hand, the Neu hydrolysate, despite of low *DH*, showed high ability in scavenging or trapping the superoxide anion, but was less efficient as ABTS scavengers, revealing that each assay reflected a different aspect of the antioxidant behavior. This could be due to different mechanisms between ABTS^•+^ and O_2_^•−^ assays as well as difference in the selectivity and reactivity of the radical species, composition of assay systems and physical location of antioxidants in the systems. The radical ABTS^•+^ may be neutralized either by direct reduction via electron transfers or by radical quenching via hydrogen atom transfer, while the O_2_^•−^ scavenging assay has not been classified into any of the mechanistic models, except for a few studies that consider that the O_2_^•−^ scavenging follows electron transfer mechanism [44]. Thus, different molecular properties of hydrolysates are of importance for specific antioxidant activity. Similar results were reported by Intrasirisawat et al. [46] who determined that the protein hydrolysate from defatted skipjack (*Katsuwonous pelamis*) roe, hydrolyzed by Alcalase exhibited different scavenging mechanisms toward ABTS, DPPH, and superoxide anion radicals. However, the hydrolysates obtained with Everlase as well as those obtained in two-step enzymatic processes with the combination of Alcalase/Flavourzyme or Neutrase/Flavourzyme exhibited high antioxidant activity in all assays, which could be associated with the presence of low molecular weight peptides. These processes seemed to be efficient in obtaining low molecular weight peptides which were commonly considered to be the most biologically active [45,46,47]. Castro and Sato [11] also found that the combined use of proteases with broad enzymatic specificities can release different peptides and thus increase the number of cleavage sites in the protein and hydrolysates, resulting in soy protein hydrolysates with higher antioxidant activity.

### 3.4. Effect of Enzymatic Hydrolysis on Sensory Properties

**Figure 3:**
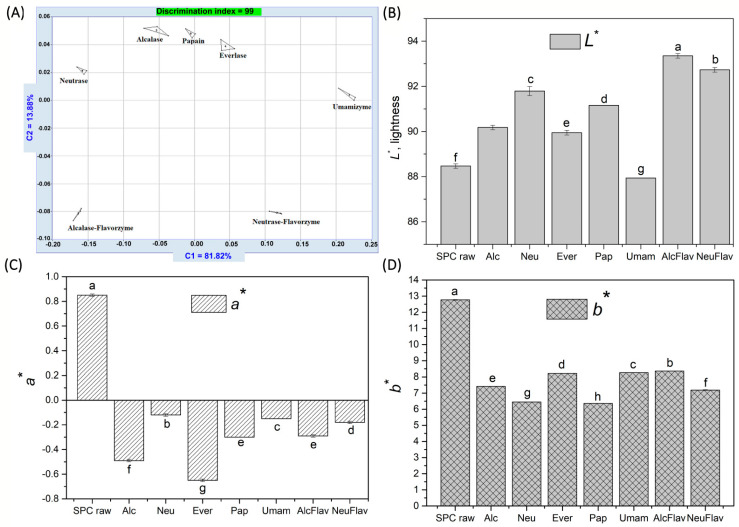
(**A**) PCA plot of hydrolysates (discrimination index = 93); color parameters of soy protein hydrolysates and SPC: (**B**) L*, lightness; (**C**) a*, red hue value; (**D**) b* yellow hue value. The results of color measuring are expressed as mean ± standard deviation (*n* = 10). Samples with different letters are significantly different from each other at level 0.05.

For soy protein hydrolysate intended for the development of functional bakery products, good taste and color are also important. Unfortunately, the enzymatic hydrolysis is often accompanied by the creation of bitter peptides and darker colors, that negatively influence the sensory properties of the hydrolysate [37,48]. The effect of enzymatic hydrolysis on bitterness and color was also examined and the results were presented in Table 2 and Figure 3.

The hydrolysis with Everlase or Neutrase resulted in higher bitterness than with other enzymes, while Umamizyme led to significantly lower bitterness even compared to R. The combination of Neutrase-Flavourzyme seemed to be the most appropriate showing improved sensory properties in terms of bitterness. Considering only the sample with lower bitterness than reference R, Friedman test ISO 8587 [49] showed that there were significant differences among them (Ftest (27.68) > Fcritic (9.37) at a 5% level of confidence). These results are in agreement with the high content of hydrophobic amino acids for the Neu hydrolysate (21.49 g/100 g protein), and lower content for the Umam (19.96 g/100 g), but they are quite surprising for the NeuFlav hydrolysate where also a high content of hydrophobic amino acids was determined (21.69 g/100 g). It is generally accepted that the bitter taste of hydrolysates is associated with the presence of low molecular weight peptides composed of mainly hydrophobic amino acids particularly leucine, proline, phenylalanine, and tyrosine [50]. However, the link between the content of these amino acids and bitterness was not always clear, suggesting that some other factors could contribute to the bitterness like specific molecular weight profile, spatial structure, the position of hydrophobic amino acid in the peptide, and bulkiness of the molecule [51]. Interestingly, after the addition of Flavourzyme and the second step, the bitterness of the Neu hydrolysate was significantly reduced, while that of the Alc even increased. This may be due to a greater tendency of Alcalase to hydrolyze at hydrophobic amino acid residues, and a higher probability of these nonpolar residues to remain at the terminus of the resulting peptides than in the Neu hydrolysate even after Flavourzyme action, causing a high bitterness.

Figure 3A shows the PCA (Principal Component Analysis) plot which provides a map of discrimination of the overall volatile composition of hydrolysates using the electronic nose system. A discrimination index of 93 was achieved between the control and hydrolysates, which explained the very distinct odor of the samples. All samples had different aroma compounds, but NeuFlav and Umam were more alike in the volatile composition being situated on the opposite side of the PCA plot compared to the other samples. Looking along the first component, PC1, NeuFlav was clearly separated from the AlcFlav. This is correlated with the sensory evaluation done by panelists.

The color analysis of the SPC and hydrolysates are given in Figure 3B–D. During hydrolysis, a significant increase in L* value and a decrease in b* value was apparent for all hydrolysates, compared with SPC. In fact, the hydrolysate AlcFlav showed the highest lightness. Regarding a* value, all samples showed negative values, with exception of the SPC. Thus, even visually seemed to be clear that the SPC sample was browner, which is equivalent to a combination of positive a* (red hue) and b* (yellow hue) values. The hydrolysate Umam was significantly darker (lower L* value) and yellower (higher b* value) than control and other hydrolysates, which impeded its utilization in food products. The color variation from the darkness to whiteness (based on the value of L* where L* = 0 is black and L* = 100 is white) was: Umam < SPC < Ever < Alc < Pap < Neu < NeuFlav < AlcFlav.

### 3.5. Rheological Characterization of Dough with Hydrolysates by Mixolab

The wheat flour fortification with soybean hydrolysates seemed to have beneficial nutritional and biological effects, but they can modify dough rheological properties, and the technological quality of bakery products. The effects of the hydrolysate addition on rheological properties by Mixolab parameters are summarized in Table 3.

The addition of different hydrolysates in the wheat flour appeared to result in significant differences in water absorption and viscoelastic properties of dough. The addition in all cases caused decrease in water absorption and increase in development time, revealing a weakening of the gluten network. Regarding stability, only the Umam caused the increase in stability from 7.79 ± 0.02 to 8.23 ± 0.06 min, revealing that the hydrolysate addition increased the time during which the dough maintained maximum consistency, whilst the addition of the Neu did not significantly influence the stability compared to control. The Pap addition resulted in the lowest dough stability. All samples showed a lower cooking stability range (C4 and C3 values), but also lower C5 values revealing lower starch retrogradation tendency. The Pap hydrolysate decreased the rate of starch retrogradation by around 32%, indicating the strong ability to delay staling of bakery products, as the retrogradation was often linked to the increased firming during prolonged storage. The dough with the NeuFlav hydrolysate showed the lowest capacity for starch gelatinization and water absorption, but the stability was rather high, making it still suitable for baking.

### 3.6. Fortification of Cookie with Soy Protein Hydrolysates

The aim of this part of the research is to develop a functional bakery product (cookie) using the selected soy protein hydrolysates as ingredients. Table 4 and Figure S2 (Supplementary materials) show the comparative results of chemical and amino acid composition, respectively, of traditional cookies with wheat flour (control) and the cookie with added Pap, Umam, and NeuFlav hydrolysates.

**Figure 4:**
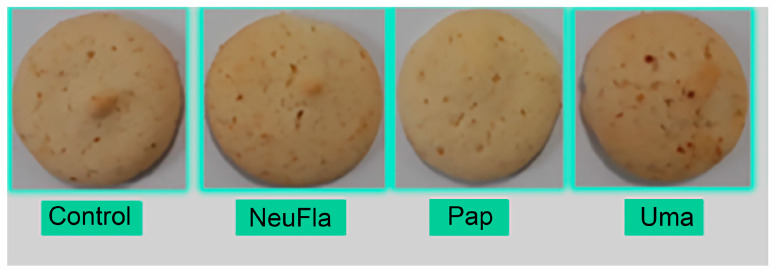
Photographic views of traditional cookies with wheat flour (control) and the cookie with added Pap, Umam, and NeuFlav hydrolysates.

The protein content of cookie prepared with hydrolysates increased by around 20% compared to control. By adding the NeuFlav hydrolysate to cookie, the content of EAA was increased from 35.36 to 38.39 g/100 g protein (Supplementary materials, Table S1). There were no significant differences between control and NeuFlav cookies in terms of appearance, taste, and overall appreciation (Figure 4).

With help of the electronic nose system, a discrimination index of 93 was achieved between control and tested cookies which explained a very distinct odor of samples as shown in Supporting information (Figure S2). The NeuFlav and Umam cookies were more alike in the volatile composition being situated on the opposite side of the PCA plot compared to the Pap cookie. Regarding the taste, cookies containing NeuFlav and Pap had lower bitterness intensity than Umam. The consumers have a consumption intention of more than 80% for the sample with the NeuFlav (data not presented). Overall, considering the nutritional quality, protein content, sensory properties, and antioxidant activity combined, cookies with 5% NeuFlav seemed to be the best new functional bakery product.

## 4. Conclusions

Several commercial proteases were examined for their ability to produce hydrolysates from SPC as a raw material with lower commercial value that is advantageous from an economic point of view. The type of protease significantly affected the kinetics of SPC hydrolysis as well as protein recovery, nutritional, sensory, and rheological properties of the hydrolysates. The enzymatic hydrolysis in all cases resulted in increase in the antioxidant activity of hydrolysate which was confirmed by three methods and all hydrolysates showed significantly improved nutrition quality compared to SPC, particularly the NeuFlav hydrolysate. The two-step enzymatic process with Neutrase and Flavourzyme seemed to be also the most appropriate enzyme combination concerning organoleptic-low bitter taste. The dough prepared with the NeuFlav had acceptable rheological properties, and resulting cookie was appreciated for the nutritional quality and consumer`s acceptability. Thus, this hydrolysate would constitute a promising ingredient in the manufacture of new functional bakery product.

## Figures and Tables

**Table 1:** Protein content, protein recovery and total amino acid content (g/100 g protein) in control sample (SPC) and hydrolysates obtained with different proteases. Results are expressed as means ± standard deviation (*n* = 3).

	SPC	Alc	Neu	Ever	Umam	Pap	Alc-Flav	Neu-Flav	FAO Reference Protein [40]
Protein content, % dw	70.96	83.07	81.25	82.17	81.29	84.91	82.85	80.23	
Protein recovery, %	-	69.39	72.04	75.93	68.59	78.17	82.19	68.24	
Amino acid content, g/100 g protein									
Arginine	6.49 ± 0.06	9.33 ± 0.09	10.27 ± 0.03	7.13 ± 0.05	6.50 ± 0.11	6.50 ± 0.07	8.52 ± 0.04	6.32 ± 0.09	
L-Lysine	6.54 ± 0.08	7.69 ± 0.12	6.84 ± 0.09	7.31 ± 0.07	9.59 ± 0.15	9.77 ± 0.14	8.56 ± 0.10	8.66 ± 0.13	5.8 (6.6)
L-Alanine	5.19 ± 0.10	3.99 ± 0.04	4.11 ± 0.15	4.27 ± 0.14	3.90 ± 0.08	4.35 ± 0.20	4.40 ± 0.12	4.29 ± 0.010	
L-Threonine	3.58 ± 0.12	3.89 ± 0.09	3.99 ± 0.14	4.20 ± 0.16	3.73 ± 0.15	4.02 ± 0.08	4.09 ± 0.07	4.05 ± 0.11	3.4 (4.3)
Glycine	3.97 ± 0.16	3.85 ± 0.11	3.85 ± 0.10	3.99 ± 0.05	3.83 ± 0.08	4.30 ± 0.09	3.99 ± 0.11	4.02 ± 0.13	
L-Valine	4.45 ± 0.17	4.02 ± 0.12	4.40 ± 0.15	4.00 ± 0.14	3.86 ± 0.09	4.63 ± 0.10	4.72 ± 0.08	4.95 ± 0.06	3.5 (5.5)
L-Serine	5.04 ± 0.08	5.34 ± 0.09	5.38 ± 0.06	5.70 ± 0.04	5.38 ± 0.10	5.27 ± 0.09	5.51 ± 0.11	5.56 ± 0.10	
L-Proline	5.29 ± 0.13	5.57 ± 0.06	5.40 ± 0.11	5.59 ± 0.10	5.70 ± 0.09	5.91 ± 0.12	5.49 ± 0.015	5.72 ± 0.08	
L-Isoleucine	4.73 ± 0.09	4.23 ± 0.12	4.49 ± 0.05	4.16 ± 0.04	4.21 ± 0.07	4.45 ± 0.09	4.75 ± 0.11	5.09 ± 0.12	2.8 * (4.6)
L-Leucine	7.71 ± 0.16	7.10 ± 0.07	7.46 ± 0.06	7.06 ± 0.08	6.57 ± 0.18	6.78 ± 0.15	7.56 ± 0.09	7.57 ± 0.07	6.6(9.3)
L-Methionine	1.67 ± 0.11	1.25 ± 0.08	1.31 ± 0.06	1.31 ± 0.09	1.24 ± 0.06	1.35 ± 0.07	1.43 ± 0.05	1.34 ± 0.10	2.5 * (4.2)
L-Histidine	2.61 ± 0.08	2.74 ± 0.06	2.50 ± 0.06	2.67 ± 0.06	2.48 ± 0.06	2.41 ± 0.06	2.41 ± 0.06	2.58 ± 0.06	1.90 (2.6)
L-Phenylalanine	4.34 ± 0.07	4.71 ± 0.09	4.84 ± 0.10	4.95 ± 0.14	4.71 ± 0.09	4.95 ± 0.14	5.31 ± 0.16	4.89 ± 0.07	6.3 ** (7.2)
L-Glutamic acid	20.46 ± 0.12	19.85 ± 0.14	20.03 ± 0.09	21.07 ± 0.07	19.32 ± 0.05	17.27 ± 0.12	16.53 ± 0.11	16.42 ± 0.10	
L-Aspartic acid	10.99 ± 0.16	11.25 ± 0.10	9.70 ± 0.14	11.17 ± 0.06	13.86 ± 0.13	12.47 ± 0.11	11.22 ± 0.08	13.39 ± 0.09	
Cystine	1.68 ± 0.13	1.61 ± 0.05	1.64 ± 0.06	2.08 ± 0.11	2.13 ± 0.12	1.92 ± 0.09	1.71 ± 0.08	1.64 ± 0.05	
L-Tyrosine	4.33 ± 0.06	3.57 ± 0.12	3.78 ± 0.10	3.34 ± 0.08	2.98 ± 0.14	3.65 ± 0.13	3.79 ± 0.12	3.51 ± 0.10	
ƩEAA	35.64	35.63	35.84	35.66	36.40	38.37	38.83	39.13	32.8 (44.3)

Results are expressed as means ± standard deviation (*n* = 3). AAS-amino acid score for NeuFlav hydrolysate for EAA: Lys: 1.5; Thr: 1.2; Val: 1.3; Ile: 1.8; Leu: 1.1; L-Met + Cystine: 1.2; L-His: 1.4; L-Phe+Tyr: 1.3; * Methionine + Cystine; ** Phenylalanine + Tyrosine.

**Table 2:** The classification of SPC hydrolysates according to the sum of the classes.

Intensity of Bitterness Lower than Reference, *R*					Intensity of Bitterness Higher than Reference, *R*	
−25	−22	−19	−15	−7	14	18
NeuFlav	Umam	Pap	Alc	AlcFlav	Neu	Ever

**Table 3:** Rheological characterization of dough with added soy protein hydrolysates obtained with different proteases assessed by Mixolab.

Sample	Water Absorption (%)	Development Time (min)	Stability (min)	C2 (Nm)	C3 (Nm)	C4 (Nm)	C5 (Nm)
Control	59.53 ± 0.06 ^a^	1.42 ± 0.02 ^d^	7.79 ± 0.02 ^b^	0.39 ± 0.01 ^a^	1.90 ± 0.02 ^a^	1.69 ± 0.01 ^a^	2.41 ± 0.02 ^a^
Alc	54.60 ± 0 ^b^	4.42 ± 0.02 ^b^	6.80 ± 0.22 ^d^	0.23 ± 0.01 ^d^	1.62 ± 0.01 ^c^	1.54 ± 0.01 ^c^	2.19 ± 0.02 ^c^
Ever	54.77 ± 0.06 ^b^	4.44 ± 0.05 ^b^	6.97 ± 0.03 ^d^	0.30 ± 0.01 ^b^	1.59 ± 0.01 ^cd^	1.51 ± 0.01 ^cd^	2.26 ± 0.01 ^b^
Neu	54.60 ± 0 ^b^	4.00 ± 0.07 ^c^	7.52 ± 0.12 ^bc^	0.25 ± 0.01 ^c^	1.41 ± 0.01 ^e^	1.26 ± 0.01 ^e^	2.02 ± 0.03 ^e^
Pap	53.17 ± 0.29 ^f^	3.88 ± 0.03 ^c^	6.33 ± 0.06 ^e^	0.20 ± 0.01 ^e^	1.06 ± 0.04 ^f^	1.27 ± 0.01 ^e^	1.87 ± 0.01 ^f^
Umam	53.87 ± 0.12 ^d^	4.72 ± 0.03 ^a^	8.23 ± 0.06 ^a^	0.30 ± 0.01 ^b^	1.55 ± 0.01 ^d^	1.47 ± 0.01 ^d^	2.10 ± 0.01 ^d^
AlcFlav	53.50 ± 0 ^e^	4.72 ± 0.14 ^a^	7.32 ± 0.10 ^c^	0.20 ± 0.01 ^e^	1.69 ± 0.04 ^b^	1.61 ± 0.03 ^b^	2.40 ± 0.04 ^a^
NeuFlav	53.0 ± 0 ^f^	4.70 ± 0.07 ^a^	7.42 ± 0.10 ^c^	0.19 ± 0.01 ^e^	1.11 ± 0.01 ^f^	1.49 ± 0.02 ^d^	2.20 ± 0.02 ^bc^

Results are expressed as means ± standard deviation (*n* = 3). Means with different letters in the same column are significantly different (*p* < 0.05).

**Table 4:** Chemical analysis of the control cookies and cookies enriched with three selected hydrolysates (% dry matter).

Sample	Control	Enriched with Pap	Enriched with NeuFla	Enriched with Umam
Protein	7.72 ± 0.03 ^d^	9.07 ± 0.02 ^bc^	9.28 ± 0.04 ^ab^	9.35 ± 0.12 ^a^
Fat	34.86 ± 0.17 ^a^	35.03 ± 0.15 ^a^	35.34 ± 0.15 ^a^	35.35 ± 0.22 ^a^
Ash	0.34 ± 0.01 ^b^	0.44 ± 0.03 ^a^	0.49 ± 0 ^a^	0.44 ± 0.01 ^a^
Sugar	17.71 ± 0.015 ^a^	17.97 ± 0.02 ^a^	17.97 ± 0.07 ^a^	18.15 ± 0.19 ^a^
Energy *	554.76 ± 0.84 ^a^	550.81 ± 0.59 ^b^	551.81 ± 0.71 ^ab^	545.67 ± 1.0 ^c^

Results are expressed as mean ± standard deviation (*n* = 2). Values followed by different letters in the same row are significantly different (*p* < 0.05). * Expressed as kcal/100 g product.

## Data Availability

The data presented in this study are available from the corresponding author upon request.

## Supplementary Materials

The following supporting information can be downloaded at: https://www.mdpi.com/article/10.3390/foods12010024/s1. Figure S1: The solubility of soy protein hydrolysates obtained with different enzymes at different pH. Figure S2: PCA plot of traditional cookie with wheat flour (control) and the cookie with added Pap, Umam and NeuFlav hydrolysates.Table S1: Comparative results of amino acid composition of traditional cookie with wheat flour (control) and the cookie with added Pap, Umam and NeuFlav hydrolysates. Results are expressed as means ± standard deviation (n = 2).
