# Supplementary material for: Impact of Different Enzymatic Processes on Antioxidant, Nutritional and Functional Properties of Soy Protein Hydrolysates Incorporated into Novel Cookies
Source: Foods. 2022 Dec 21;12(1):24. doi: 10.3390/foods12010024 (PMC9818677; doi:10.3390/foods12010024)
Supplement: Supplementary file 1 [file foods-12-00024-s001.zip › Supplementary Materials.pdf]

Supplementary Materials: Supporting information may be found in the online version of this article (Table S1, Figures S1 and S2).

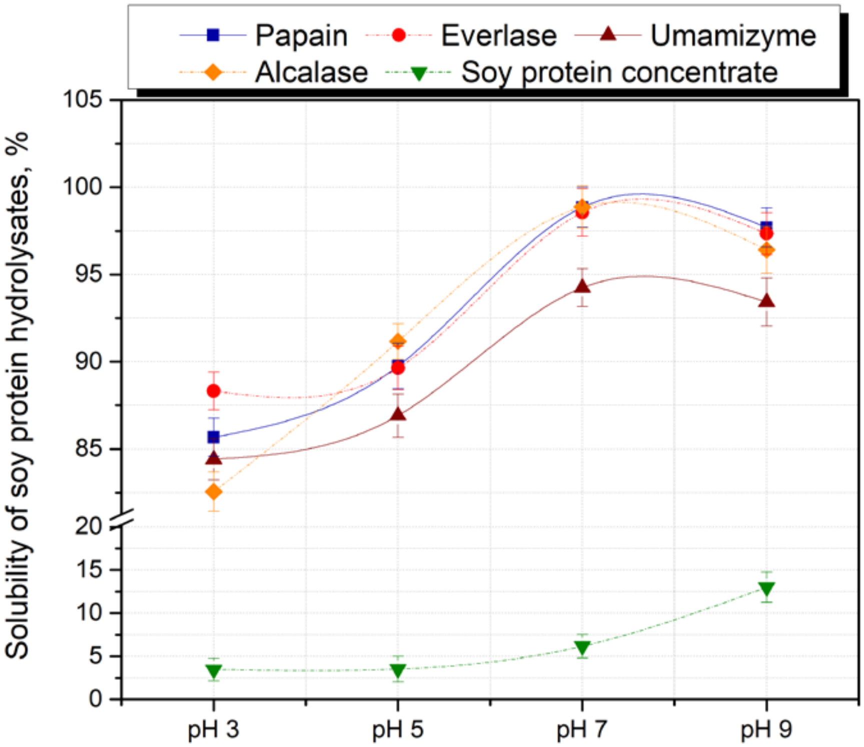

Figure S1: The solubility of soy protein hydrolysates obtained with different enzymes at different pH.

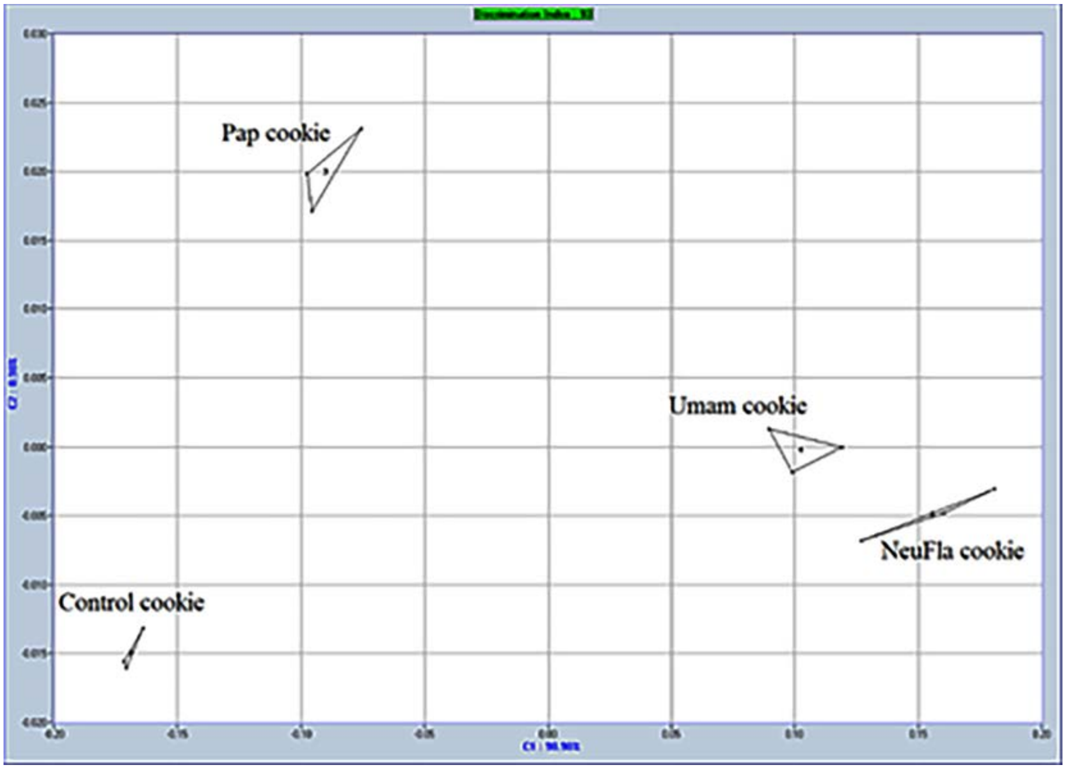

Figure S2: PCA plot of traditional cookie with wheat flour (control) and the cookie with added Pap, Umam and NeuFlav hydrolysates.

Table S1: Comparative results of amino acid composition of traditional cookie with wheat flour (control) and the cookie with added Pap, Umam and NeuFlav hydrolysates. Results are expressed as means ± standard deviation (*n* = 2).

	**Amino acid content, g/100 g**			
	**SPC**	**Pap**	**Umam**	**NeuFlav**
Arginine	9.90±0.08	11.3±0.11	10.6±0.09	10.9±0.08
Lysine	6.33±0.04	7.15±0.14	7.72±0.10	7.66±0.12
Alanine	6.74±0.06	9.09±0.10	4.91±0.15	6.10±0.08
Threonine	4.40±0.04	4.73±0.11	4.44±0.08	4.66±0.10
Glycine	4.13±0.04	4.24±0.06	4.09±0.06	4.22±0.06
Valine	4.95±0.04	4,85±0.12	5.73±0.10	5.10±0.09
Serine	6.88±0.08	7.03±0.09	6.78±0.10	7.10±0.10
Proline	11.00±0.04	10.18±0.09	10.64±0.10	10.76±0.08
Isoleucine	3.99±0.04	4.48±0.09	5.03±0.04	4.44±0.10
Leucine	8.67±0.13	8.73±0.11	9.24±0.13	8.88±0.80
Methionine	1.65±0.06	1.94±0.06	0.82±0.08	1.55±0.06
Histidine	1.38±0.06	2.06±0.10	1.87±0.06	2.00±0.06
Phenylalanine	3.99±0.08	4.00±0.11	4.09±0.04	4.10±0.04
Glutamic acid	18.70±0.05	13.45±0.12	16.14±0.18	15.32±0.08
Aspartic acid	3.58±0.04	3.15±0.04	4.56±0.11	4.11±0.05
Cystine	1.24±0.04	1.33±0.02	0.82±0.04	0.78±0.09
Tyrosine	2.48±0.11	2.30±0.14	2.46±0.20	2.33±0.30

ΣEAA	35.36	37.94	38.94	38.39
